# Machine Learning Integrating SERF-MEG and VEP for Diagnosis and Differential Diagnosis of Optic Neuropathies

**DOI:** 10.3390/bioengineering13080885

**Published:** 2026-07-31

**Authors:** Helei Wang, Yuankun Qi, Yu Lou, Xu Zhang, Xinda Song

**Affiliations:** 1 School of Instrumentation and Optoelectronic Engineering, Beihang University, Beijing 100191, China; wanghelei326@buaa.edu.cn (H.W.);; 2 Hangzhou National Institute of Extremely-Weak Magnetic Field Infrastructure, Hangzhou 310051, China; 3 State Key Laboratory of Traditional Chinese Medicine Syndrome, Hangzhou 310051, China

**Keywords:** SERF-MEG, optic neuropathy, VEP, machine learning, differential diagnosis, explainable artificial intelligence

## Abstract

Visual evoked potential (VEP) is widely used to assess optic nerve function, but its ability to differentiate optic neuropathies remains limited. This study investigated the diagnostic value of spin-exchange relaxation-free magnetoencephalography (SERF-MEG), alone and in combination with VEP, using machine learning. A total of 142 eyes, including 71 healthy controls (HC), 34 eyes with optic neuritis (ON), and 37 eyes with ischemic optic neuropathy (ION), were enrolled. Three feature sets were constructed from VEP, SERF-MEG, and their combination. Repeated stratified 5-fold nested cross-validation was used to evaluate nine supervised machine learning algorithms, and SHAP analysis was used to evaluate the interpretability of the models. The multilayer perceptron model obtained an AUC of 0.792 and an MCC of 0.470, whereas the combination VEP–MEG model performed the best in differentiating HC from optic neuropathies. The MEG model fared better than the VEP and combined models for distinguishing ON from ION, with logistic regression obtaining the greatest AUC of 0.847. Cortical network measurements and visually evoked response properties were found to contribute the most to model prediction, according to SHAP analysis. These results imply that SERF-MEG may enhance the diagnosis and differential diagnosis of optic neuropathies by offering supplementary neurophysiological data beyond traditional VEP.

## 1. Introduction

Optic neuropathy is one of the primary causes of acquired visual impairment. It may develop from inflammation, ischemia, trauma, compression, genetic diseases, or other etiologies. Among these disorders, optic neuritis (ON) and ischemic optic neuropathy (ION) are the two most common forms encountered in clinical practice [1,2]. Although they result from different pathogenic causes, these disorders commonly appear with diminished visual acuity, visual field abnormalities, dyschromatopsia, and a relative afferent pupillary deficiency [2,3]. Such overlap may obscure the underlying cause, particularly during the early stage of disease. Accurate differentiation is therefore essential because treatment strategies, prognosis, and long-term management vary substantially between these disorders [4,5].

The pathological basis of ON differs fundamentally from that of ION. ON is primarily an immune-mediated inflammatory demyelinating disorder and is more frequently observed in young and middle-aged adults. In contrast, ION is mainly caused by insufficient blood supply to the optic nerve and usually occurs in older individuals with vascular risk factors such as hypertension, diabetes mellitus, or atherosclerosis [4]. These pathological differences are expected to produce distinct functional changes along the visual pathway. However, whether conventional electrophysiological examinations can reliably capture these differences remains uncertain.

Visual evoked potentials (VEPs) are among the most widely used objective electrophysiological tests for evaluating visual pathway conduction [6]. Because tissue damage differs between ON and ION, characteristic VEP changes have also been reported. In ON, inflammatory demyelination commonly leads to prolonged P100 latency, which reflects delayed neural conduction. By comparison, ION is characterized mainly by ischemic axonal injury and loss of retinal ganglion cell axons. As a result, reduced P100 amplitude is more frequently observed, indicating a decrease in the number of functional conducting fibers [4,7,8]. On the basis of these findings, several studies have suggested that VEP parameters may assist in the differential diagnosis of optic neuropathies [9].

Nevertheless, the diagnostic performance of VEP alone remains limited. As the disease progresses, demyelination and axonal degeneration often coexist. Consequently, latency prolongation and amplitude reduction may appear in both ON and ION, leading to considerable overlap in electrophysiological findings [10,11]. In addition, VEP is essentially a scalp-recorded time-domain signal that mainly reflects the overall conduction status of the visual pathway. The physiological information obtained from this technique is therefore relatively limited.

Within the framework of the present study, attention is extended beyond conduction delay alone. Recent neuroimaging and neurophysiological studies have shown that visual dysfunction is accompanied not only by impaired optic nerve conduction but also by alterations in cortical activity and functional brain networks. Abnormal functional connectivity and adaptive network reorganization have been consistently reported, particularly in patients with ON and multiple sclerosis-related visual impairment [12,13]. These findings suggest that conventional VEP measures may not fully characterize the neural changes associated with different optic neuropathies. Additional neurophysiological information may therefore improve disease characterization and facilitate differential diagnosis.

Magnetoencephalography (MEG) is a non-invasive functional neuroimaging method that quantifies the magnetic fields generated by synchronized neural activity. It provides millisecond temporal precision and great spatial localization accuracy, rendering it ideal for investigating cortical processes in the human brain [14]. In contrast to VEP, which measures electrical potentials from the scalp, MEG identifies magnetic signals that are less influenced by the conductive properties of the skull and scalp. Consequently, cortical neuronal activity can be captured more directly and with enhanced spatial precision [15].

Recent developments in magnetic sensor technologies have considerably broadened the clinical usefulness of MEG. In particular, optically pumped magnetometers (OPMs) and spin-exchange relaxation-free (SERF) magnetometers have enabled the development of more flexible and accessible MEG systems [16]. Within this context, MEG is no longer restricted to standard measurements of response latency and amplitude. It can also characterize brain oscillations, functional connectivity, and graph-theoretical network features. These complementary assessments give objective biomarkers that indicate brain function from several viewpoints [17,18].

Evidence from neurological illnesses implies that disease-related changes often extend beyond local cortical activity. Changes in brain oscillations and functional networks have been documented in a range of neurological disorders, and many of these anomalies cannot be detected by traditional VEP alone [19,20]. Together, these findings suggest that MEG has the potential to complement conventional VEP by capturing cortical functional alterations that are difficult to detect using traditional electrophysiological measures alone.

Within the framework of the present study, visually evoked MEG signals were analyzed together with machine learning techniques to evaluate their diagnostic value in optic neuropathies. Multidimensional neurophysiological features were extracted from the time domain, frequency domain, functional connectivity, and graph-theoretical network analyses. Two binary classification tasks were designed. The first task distinguished HC from patients with optic neuropathies (ON + ION) to evaluate the overall disease detection capability. The second task differentiated patients with ON from those with ION to investigate the potential value of MEG for differential diagnosis.

To further examine the contribution of different sources of information, three feature sets were constructed, including VEP features, MEG features, and a combined VEP–MEG feature set. Their classification performance was compared systematically to determine whether multimodal information could improve diagnostic accuracy. The present study aimed to evaluate the feasibility of spin-exchange relaxation-free magnetoencephalography (SERF-MEG) as an objective neurophysiological biomarker for optic neuropathies and to provide preliminary evidence for its future clinical translation in precision diagnosis and individualized management.

## 2. Materials and Methods

### 2.1. Research Design and Ethical Authorization

**Figure 1:**
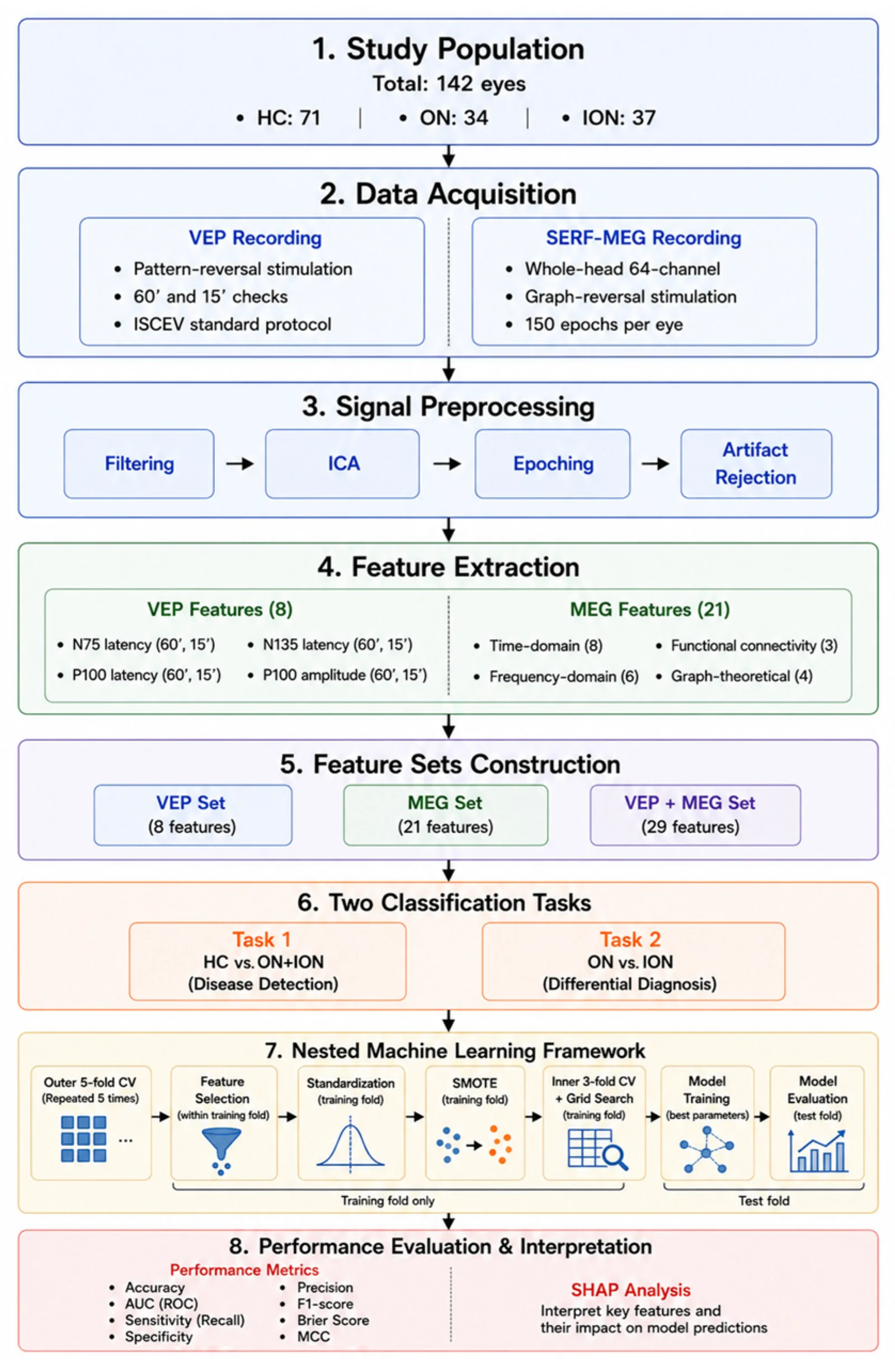
Overview of the study design and machine learning workflow. The pipeline includes study population enrollment, VEP and SERF-MEG data acquisition, signal preprocessing, feature extraction, construction of VEP, MEG, and combined feature sets, two binary classification tasks, nested machine learning analysis, and model evaluation with SHAP interpretation.

This prospective observational study evaluated the diagnostic value of VEP and MEG features for optic neuropathies. Within the framework of this study, the classification performance of individual modalities and their combined features was also compared. The overall study workflow is presented in Figure 1.

A total of 142 eyes from 142 participants were included in this study. Only one eye from each participant was included in the machine learning analysis to ensure sample independence and avoid potential information leakage caused by bilateral data from the same individual. For patients with unilateral optic neuropathy, the affected eye was selected for analysis. For patients with bilateral involvement, the eye with more severe visual impairment was selected according to clinical evaluation. The cohort consisted of 71 HC eyes, 34 eyes with ON, and 37 eyes with ION. The diagnoses of ON and ION were established according to internationally accepted neuro-ophthalmic diagnostic criteria. Clinical presentation, ophthalmic examination, visual function assessment, and relevant ancillary examinations were considered together during diagnosis. HC had no history of neurological disease, ocular structural disease, or any other condition that could affect visual function.

The exclusion criteria were as follows: (1) severe corneal, lenticular, or retinal disorders affecting visual acuity; (2) concomitant neurological diseases; (3) inability to maintain stable fixation or cooperate during the examinations; and (4) obvious artifacts or inadequate signal quality in either the MEG or VEP recordings. All participants completed standardized VEP and MEG examinations, and complete datasets were obtained for subsequent analysis. Because this study focused on an exploratory evaluation of SERF-MEG-based biomarkers and machine learning feasibility, the sample size was determined based on the availability of clinically characterized participants during the study period rather than a predefined statistical power calculation.

The study protocol was approved by the Ethics Committee of Qilu Hospital of Shandong University (approval No. KYLL-202310-017-1). The study followed the principles of the Declaration of Helsinki. Written informed consent was obtained from every participant before enrollment.

### 2.2. VEP Acquisition and Feature Extraction

VEPs were recorded using a commercial electrophysiological system (RETI-Port/Scan 21, Roland Consult, Brandenburg an der Havel, Germany). A standard pattern-reversal stimulation paradigm was applied throughout the examination. Each participant viewed a central fixation target under monocular testing conditions while full-field checkerboard stimuli were presented. Two check sizes were used: 60′ (60 min checks) and 15′ (15 min checks). The stimulation protocol and recording conditions complied with the recommendations of the International Society for Clinical Electrophysiology of Vision (ISCEV) [6]. The recorded VEP signals were processed using the routine preprocessing procedures provided by the recording system. Averaged waveforms were then generated. An experienced examiner identified all waveform components.

For each check size, the latencies of the N75, P100, and N135 components (ms) were measured. The P100 amplitude (μV) was also extracted and was defined as the peak-to-peak amplitude between the N75 and P100 components. In total, eight VEP features were obtained, including four parameters from the 60′ stimulus and four parameters from the 15′ stimulus. These features constituted the VEP feature set used for the subsequent machine learning analysis. These features were used as the conventional electrophysiological reference for all subsequent classification analyses.

### 2.3. MEG Data Acquisition and Signal Preprocessing

MEG signals were recorded using a SERF-MEG system (LMEG-64A, Zero Magnetic Medical Technology Co., Ltd., Hangzhou, China). The system was based on SERF optically pumped magnetometers and incorporated a 64-channel sensor array. The sensors covered the frontal, parietal, temporal, and occipital regions, allowing whole-brain recording of visually evoked magnetic responses. All recordings were performed using the integrated magnetic shielding and active compensation system of the MEG platform. Active magnetic field compensation was used to further reduce environmental interference. The sensor sensitivity was better than 15 fT/√Hz within the 1–30 Hz frequency range.

Before data acquisition, each participant removed all metallic objects and lay comfortably in the supine position. The head was secured inside an adult helmet, and system calibration was completed before recording. A standard pattern-reversal visual stimulation paradigm was then applied. The stimulus consisted of a black-and-white checkerboard with equal black and white areas. Pattern reversal was presented at 2.0 reversals/s, while the overall luminance remained constant throughout the experiment. Monocular stimulation was performed with the fellow eye occluded. One hundred and fifty stimulus trials were collected for each eye.

MEG preprocessing was performed in MATLAB R2020a (MathWorks, Natick, MA, USA). A 50 Hz notch filter and a 1–40 Hz band-pass filter were applied to suppress power-line interference and remove low-frequency drift and high-frequency noise. Independent component analysis (ICA) was then used to identify and remove physiological artifacts, including eye movements, eye blinks, and cardiac activity. Twenty independent components were estimated, and artifact-related components were identified by visual inspection and manually removed. After component rejection, the cleaned signals were reconstructed in the original sensor space. All 64 MEG channels were retained for subsequent analyses.

Continuous recordings were segmented into epochs according to the stimulus trigger. Each epoch extended from −100 ms to 400 ms relative to stimulus onset. Baseline correction was performed using the −100 to 0 ms pre-stimulus interval. Epochs containing obvious motion artifacts, baseline instability, or abnormal signal fluctuations were excluded. Only trials with stable waveforms and clear visually evoked responses were retained for subsequent analyses.

### 2.4. MEG Feature Extraction

#### 2.4.1. Time-Domain Features

Time-domain features were extracted from visually evoked field (VEF) signals recorded over the occipital cortex. Within the framework of this study, all valid trials from each participant were first averaged to obtain an evoked response waveform. The analysis window was defined as 70–200 ms after stimulus onset. An automatic peak detection algorithm was applied to every occipital channel. The amplitudes and latencies of the M100 and M135 components were identified automatically. The peak-to-peak (P2P) amplitude was defined as the difference between the positive and negative peaks.

Two complementary groups of time-domain features were extracted to characterize both the strongest local response and the overall cortical response. First, the occipital channel with the largest peak-to-peak amplitude was identified. Four features were extracted from this representative channel, including the M100 amplitude, M100 latency, M135 amplitude, and peak-to-peak amplitude. These measurements represented the maximal visually evoked response. The second feature group described the global response across the occipital cortex. Corresponding measurements were averaged across all valid occipital channels. Four additional features were obtained, including the mean M100 amplitude, mean M100 latency, mean M135 amplitude, and mean peak-to-peak amplitude. In total, eight time-domain MEG features were included in the subsequent machine learning analysis.

#### 2.4.2. Features of the Frequency Domain

MEG signals captured over the occipital brain were used to derive frequency-domain characteristics. Because these oscillations are strongly linked to visual cortex activity and visual pathway impairment, the investigation in this study concentrated on the alpha (7–14 Hz) and beta (14–30 Hz) frequency bands.

Spectral power throughout each frequency band was estimated using power spectral density (PSD) analysis. The proportion of the relative spectral power was estimated as the proportion of the relative spectral power. The computation was carried out as follows:

$$Relative\;Power=\frac{\sum_{f=f_{1}}^{f_{2}}PSD(f)}{\sum_{f=f_{low}}^{f_{high}}PSD(f)}$$

where PSD(f) denotes the PSD at frequency f; $f_{1}$ and $f_{2}$ identify the lower and upper bounds of the target frequency band; and $f_{low}$ and $f_{high}$ define the overall analysis range. The entire frequency range used in this investigation was 1–40 Hz. Relative power reflects the contribution of a specific frequency band to overall neural activity while reducing inter-individual variability in absolute power measurements [21].

The spatial variability of spectral activity was also evaluated. For each frequency band, the standard deviation of spectral power across all occipital channels was calculated as the power standard deviation, according to the following equation:

$$Power_{STD}\;=\;\sqrt{\frac{1}{N\;-\;1}\sum_{i=1}^{N}(P_{i}\;-\;\bar{P}{)}^{2}}$$

where $P_{i}$ denotes the spectral power of the i-th channel, $\bar{P}$ represents the mean power across all occipital channels, and N is the number of channels.

Time–frequency analysis was subsequently performed using Morlet wavelets. Stimulus-induced changes in oscillatory activity were quantified, and the mean time–frequency power within the alpha and beta bands was calculated across the occipital cortex. These measurements characterized the dynamic modulation of neural oscillations following visual stimulation.

A total of six frequency-domain features were extracted for the subsequent machine learning analysis. These features included alpha relative power, beta relative power, alpha power standard deviation, beta power standard deviation, alpha time–frequency power, and beta time–frequency power.

#### 2.4.3. Functional Connectivity Features

Functional connectivity analysis was performed to evaluate neural synchronization and information exchange between different brain regions. Connectivity measures were calculated over the entire visually evoked response period and were summarized within a predefined occipital region of interest (ROI).

Linear functional coupling between MEG channels was first estimated using the Pearson correlation coefficient. The calculation was defined as follows:

$$r_{xy}\;=\;\frac{\sum_{i=1}^{N}(x_{i}\;-\;\bar{x})(y_{i}\;-\;\bar{y})}{\sqrt{\sum (x_{i}\;-\;\bar{x}{)}^{2}\sum (y_{i}\;-\;\bar{y}{)}^{2}}}$$

where $x_{i}$ and $y_{i}$ represent the signals recorded from two MEG channels, and $\bar{x}$ and $\bar{y}$ denote their corresponding mean values.

The Pearson correlation coefficient was calculated for every channel pair within the occipital ROI. The average correlation coefficient was then computed and used as the mean Pearson functional connectivity, representing the overall strength of functional coupling within the visual cortex.

To reduce the influence of volume conduction and common signal sources, the weighted phase lag index (wPLI) was further calculated to quantify phase synchronization between brain regions. The definition of wPLI is given below:

$$wPLI\;=\;\frac{|E(|Im(X)|\cdot sign(Im(X)))|}{E(|Im(X)|)}$$

where Im(X) represents the imaginary part of the cross-spectrum between two MEG channels, and E(⋅) denotes the expectation operator. Higher wPLI values indicate stronger non-zero-lag phase synchronization between brain regions while being less susceptible to the effects of volume conduction and common signal sources [22].

Mean wPLI values were calculated separately for the alpha (7–14 Hz) and beta (14–30 Hz) frequency bands. In total, three functional connectivity features were obtained, including the mean Pearson functional connectivity, the mean alpha-band wPLI, and the mean beta-band wPLI.

#### 2.4.4. Graph-Theoretical Features

Within the framework of this study, graph theory was applied to characterize the organization of brain functional networks during visual stimulation. Functional networks were constructed from whole-brain 64-channel MEG recordings using the wPLI in the alpha band (7–14 Hz). Each MEG channel was defined as a network node, whereas the functional connection between two channels was defined as a network edge. The connectivity matrix was then binarized using an absolute wPLI threshold of 0.10, where connections with wPLI values ≥0.10 were retained as network edges, and an undirected graph was generated for subsequent network analysis.

Four widely used graph-theoretical metrics were calculated to quantify different aspects of network organization.

Global efficiency was used to evaluate the overall capacity of the network for information integration. It was calculated as:

$$E_{global}\;=\;\frac{1}{N(N\;-\;1)}\sum_{i\neq j}\frac{1}{L_{ij}}$$

where $L_{ij}\;$represents the shortest path length between nodes i and j.

Local efficiency was calculated to assess information transfer within local subnetworks:

$$E_{local}\;=\;\frac{1}{N}\sum_{i=1}^{N}E_{global}(G_{i})$$

where $G_{i}$ denotes the subnetwork formed by the immediate neighbors of node i.

Clustering coefficient was used to describe the tendency of neighboring nodes to form locally interconnected clusters. It was calculated as:

$$C_{i}\;=\;\frac{2e_{i}}{k_{i}(k_{i}\;-\;1)}$$

where $e_{i}\;$is the number of existing connections among the neighbors of node i, and $k_{i}\;$denotes the degree of that node.

Mean degree reflects the average number of connections across all network nodes. The degree of each node was defined as

$$Degree_{i}=\sum_{j}A_{ij}$$

where $A_{ij}\;$is the corresponding element of the adjacency matrix. The mean degree of the network was then calculated as

$$Mean\;Degree=\frac{1}{N}\sum_{i=1}^{N}Degree_{i}$$

These four graph-theoretical metrics were included as MEG features for machine learning analysis. In the context of this study, they were used to characterize alterations in functional network organization and connectivity associated with optic neuropathies. Their definitions and computational procedures followed established methods in brain network analysis [23].

### 2.5. Machine Learning Analysis

#### 2.5.1. Feature Set Construction

Within the framework of this study, three feature sets were created to evaluate the diagnostic value of VEP and SERF-MEG for optic neuropathies. The VEP feature set included eight parameters obtained under the 60′ and 15′ checkerboard stimulation conditions. The MEG feature set consisted of multidimensional neurophysiological features extracted from time-domain responses, frequency-domain analysis, functional connectivity, and graph-theoretical network analysis. The combined feature set incorporated all VEP and MEG features. Two binary classification tasks were designed according to the study objectives. The first task distinguished HC from patients with optic neuropathies (ON + ION) and was used to evaluate the overall disease detection performance. The second task differentiated ON from ION and was used to assess differential diagnostic performance.

Feature selection was performed independently within each outer cross-validation training fold to eliminate feature redundancy and minimize overfitting. Random Forest feature importance and the absolute coefficients of L1-regularized logistic regression were initially calculated. These two importance measures were standardized using Z-score normalization and combined to obtain a composite importance score. This strategy was designed to integrate complementary feature-ranking information: Random Forest importance can capture nonlinear relationships between features and classification outcomes, whereas L1-regularized logistic regression provides sparse selection of potentially informative variables. Equal weighting was adopted because no prior evidence supported assigning greater importance to either feature-ranking method. Features were ranked according to the composite score. Minimum quotas were predefined for each MEG feature domain, including time-domain, frequency-domain, functional connectivity, and graph-theoretical features, to ensure representation of different neurophysiological information. For the HC versus ON + ION classification task, the minimum quotas were set as two time-domain features, two frequency-domain features, one functional connectivity feature, and one graph-theoretical feature. For the ON versus ION classification task, the corresponding minimum quotas were set as one feature from each MEG feature domain. Feature selection was performed exclusively within the current training fold, whereas the corresponding test fold remained completely unseen throughout the process to prevent information leakage [24]. The robustness of the feature selection strategy was further evaluated by comparing RF-only, L1-only, and hybrid selection approaches. The detailed comparison results are provided in Supplementary Table S1.

Based on this procedure, the HC versus ON + ION task retained eight VEP features, twelve MEG features, and fourteen combined features (five VEP and nine MEG features). The ON versus ION task retained eight VEP features, eight MEG features, and ten combined features (three VEP and seven MEG features) for subsequent model development.

#### 2.5.2. Model Construction and Validation

All machine learning analyses were performed in Python (version 3.10.20) using the Scikit-learn, XGBoost, and LightGBM libraries [25,26,27]. Because class imbalance was present in both classification tasks, continuous variables were first standardized using Z-score normalization. The mean and standard deviation were estimated only from the current outer training fold, and the same transformation was subsequently applied to the corresponding test fold. The Synthetic Minority Over-sampling Technique (SMOTE) was then applied only to the standardized training data to balance class distributions before model training [28].

Nine supervised learning algorithms were evaluated, including Logistic Regression, Support Vector Machine with a radial basis function kernel (SVM-RBF), Random Forest, Gradient Boosting, Extra Trees, AdaBoost, Multi-layer Perceptron (MLP), Extreme Gradient Boosting (XGBoost), and Light Gradient Boosting Machine (LightGBM). Model performance was evaluated using repeated stratified five-fold cross-validation with five repetitions, resulting in 25 independent train–test splits. This design retained class proportions in every fold while decreasing the unpredictability produced by a single data division. Within each outer training fold, feature selection, data standardization, SMOTE, hyperparameter tuning, and model fitting were performed only on the training data. The appropriate test fold was reserved purely for final performance evaluation, thereby limiting information loss and boosting the reliability of model assessment [29].

For models with tunable hyperparameters, grid search combined with three-fold stratified cross-validation was performed exclusively within the training fold. The optimal hyperparameter combination was selected according to the mean validation Area Under the Receiver Operating Characteristic Curve (AUC) [25]. The predefined search spaces for each classifier, including the evaluated parameter ranges and selected optimal hyperparameters, are provided in Supplementary Table S2A,B. For each classification task and each feature set, the model with the highest mean outer-fold AUC was selected as the final model. Shapley additive explanations (SHAP) analysis was subsequently performed to quantify the contribution of individual features to model predictions.

#### 2.5.3. Performance Evaluation

Model performance was evaluated using Accuracy, AUC, F1-score, Sensitivity, Specificity, Precision, the Brier Score, and the Matthews Correlation Coefficient (MCC). AUC was used to assess overall discriminative performance. F1-score summarized the balance between precision and recall. The Brier Score evaluated the calibration of predicted probabilities. MCC was selected as an additional overall performance metric because it simultaneously considers true positives, true negatives, false positives, and false negatives and remains robust in the presence of class imbalance [30,31]. All performance metrics are reported as the mean ± standard deviation (mean ± SD) across the 25 outer test folds. These metrics were used to compare the classification performance across feature sets and machine learning algorithms. Pairwise comparisons between models were performed using paired bootstrap analysis based on out-of-fold predictions from identical cross-validation splits. AUC differences were calculated over 5000 bootstrap iterations, and 95% confidence intervals and two-sided *p* values were obtained. To further evaluate model stability under the limited sample size, bootstrap resampling was performed to estimate confidence intervals of AUC values.

## 3. Results

### 3.1. Clinical Characteristics of Study Population

The study population consisted of 142 participants, including 71 healthy controls, 34 patients with optic neuritis, and 37 patients with ischemic optic neuropathy. Demographic and clinical characteristics of the study population, including age, sex, visual acuity, and previous treatment status, are summarized in Table 1.

### 3.2. Classification of HC and Patients with Optic Neuropathies

Within the framework of this study, three feature sets were evaluated to assess the diagnostic performance of VEP and SERF-MEG for identifying optic neuropathies. Separate machine learning models were developed using the VEP feature set, the MEG feature set, and the combined VEP–MEG feature set. Nine supervised learning algorithms were evaluated, including Logistic Regression, SVM-RBF, Random Forest, Gradient Boosting, Extra Trees, AdaBoost, MLP, XGBoost, and LightGBM. The detailed results are summarized in Table 2. Across all models, the combined feature set generally achieved the best classification performance. The VEP feature set performed slightly better than the MEG feature set overall, whereas integrating VEP and MEG features further improved model performance, indicating that the two modalities provided complementary diagnostic information.

**Figure 2:**
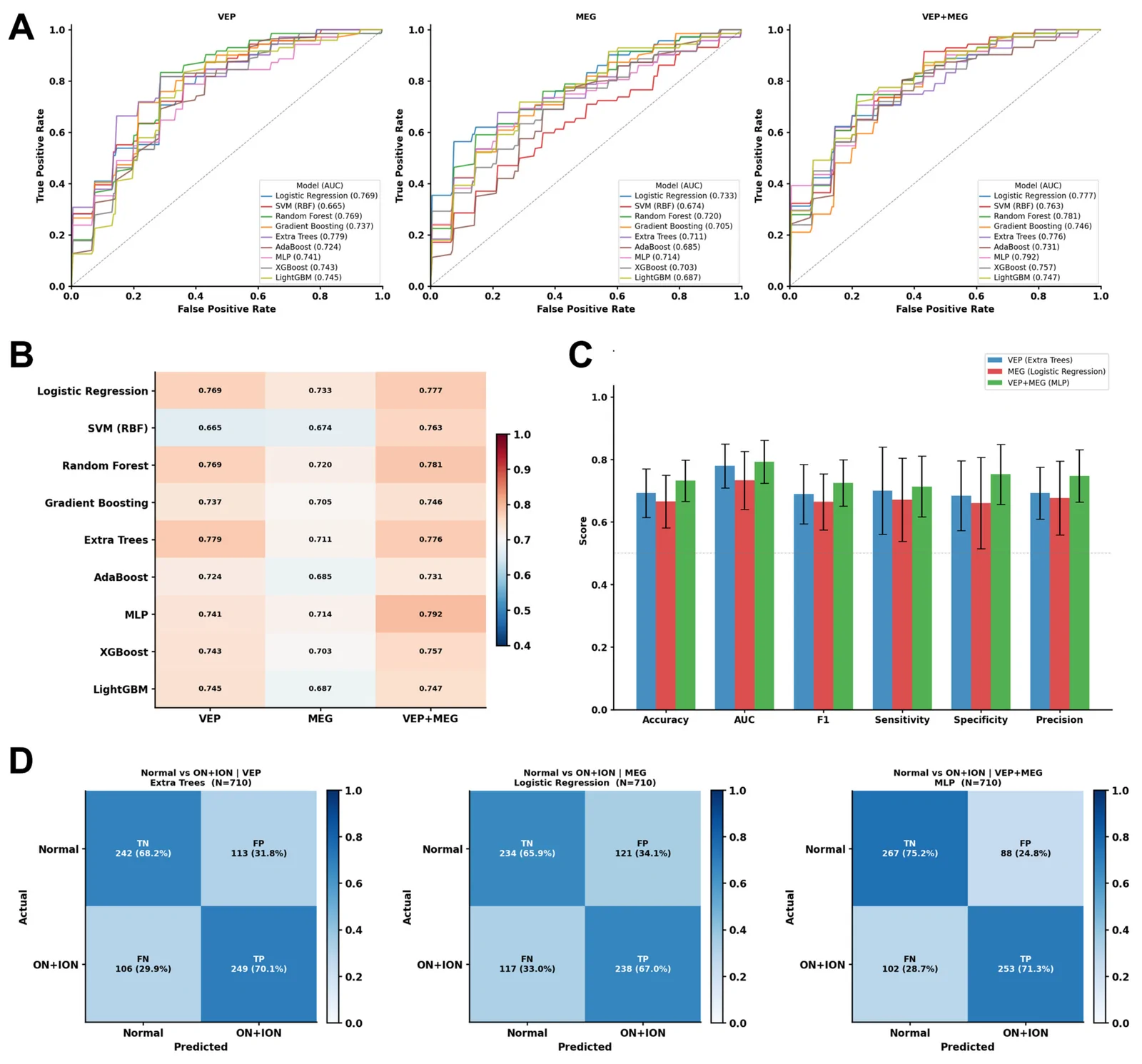
Performance comparison of machine learning models for HC versus ON + ION classification. (**A**) Receiver operating characteristic (ROC) curves of nine machine learning algorithms using the VEP, MEG, and combined VEP + MEG feature sets. The area under the ROC curve (AUC) is reported for each classifier. (**B**) Heatmap summarizing the AUC values of all evaluated classifiers across the three feature sets. (**C**) Comparison of the best-performing models from each feature set using six classification metrics (Accuracy, AUC, F1-score, Sensitivity, Specificity, and Precision); error bars represent the standard deviation across repeated outer cross-validation folds. (**D**) Confusion matrices of the best-performing models based on the cumulative predictions from all outer test folds during repeated stratified 5-fold cross-validation.

As shown in Figure 2, the VEP feature set produced relatively stable classification performance across different machine learning algorithms. The Extra Trees model achieved the highest AUC (0.779 ± 0.070), whereas the Random Forest model yielded the highest accuracy (0.708 ± 0.083) and a relatively high MCC (0.427 ± 0.172). These results indicate that conventional VEP features can effectively distinguish HC from patients with optic neuropathies.

The MEG feature set achieved slightly lower overall performance than the VEP feature set. Logistic Regression produced the highest AUC (0.733 ± 0.093), while the MLP model achieved the highest accuracy (0.675 ± 0.090) and MCC (0.359 ± 0.186). These findings suggest that MEG features alone provide moderate diagnostic performance for disease identification.

Performance was further improved after VEP and MEG features were combined. Among all models, MLP achieved the best overall performance, with an accuracy of 0.732 ± 0.066, an AUC of 0.792 ± 0.069, an F1-score of 0.725 ± 0.074, and an MCC of 0.470 ± 0.133. Random Forest and Extra Trees also performed consistently well, achieving AUC values of 0.781 ± 0.070 and 0.776 ± 0.080, respectively.

Overall, the combined feature set showed the highest numerical performance across the major evaluation metrics, including Accuracy, AUC, and MCC. Compared with the best VEP-based model, the combined model increased the AUC from 0.779 to 0.792 and improved the MCC from 0.427 to 0.470. To further evaluate whether these performance differences were statistically significant, paired bootstrap AUC comparisons were performed using out-of-fold predictions generated from identical cross-validation splits. The improvement in the VEP–MEG model over the VEP-only model did not reach statistical significance (ΔAUC = 0.017, 95% CI: −0.017 to 0.052, *p* = 0.302), whereas the VEP–MEG model showed a significant improvement compared with the MEG-only model (ΔAUC = 0.058, 95% CI: 0.017 to 0.101, *p* = 0.004). Detailed pairwise bootstrap comparisons of AUC differences are provided in Supplementary Table S3.

### 3.3. Classification of ON and ION

A second classification task was conducted to evaluate the ability of SERF-MEG to differentiate between ON and ION. Separate models were developed using the VEP, MEG, and combined feature sets. The detailed results are presented in Table 3.

**Figure 3:**
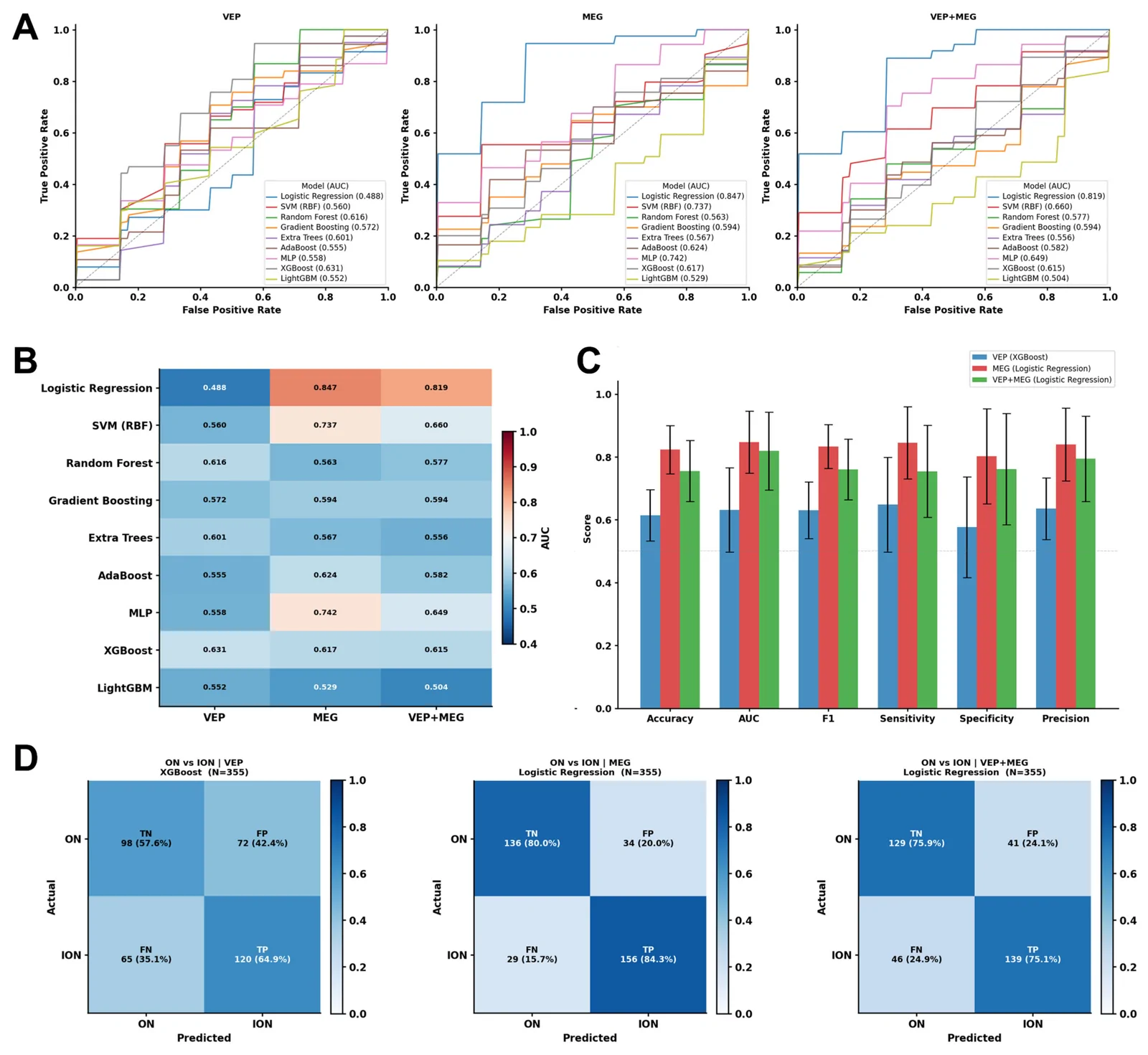
Performance comparison of machine learning models for ON versus ION classification. (**A**) Receiver operating characteristic (ROC) curves of nine machine learning algorithms using the VEP, MEG, and combined VEP + MEG feature sets. The AUC of each classifier is indicated in the legend. (**B**) Heatmap summarizing the AUC values obtained by all evaluated classifiers across the three feature sets. (**C**) Performance comparison of the best-performing models from each feature set using six classification metrics (Accuracy, AUC, F1-score, Sensitivity, Specificity, and Precision); error bars indicate the standard deviation across repeated outer cross-validation folds. (**D**) Confusion matrices of the best-performing models based on the cumulative predictions from all outer test folds during repeated stratified 5-fold cross-validation.

Compared with the HC versus ON + ION task, distinguishing ON from ION was considerably more challenging. Overall, the VEP feature set showed limited discriminative ability across most machine learning algorithms. As shown in Figure 3, XGBoost achieved the best performance among the VEP-based models, with an accuracy of 0.614 ± 0.082, an AUC of 0.631 ± 0.134, and an MCC of 0.236 ± 0.174. The remaining models produced AUC values close to random classification, indicating that conventional VEP features alone had limited value for differentiating ON from ION.

In contrast, the MEG feature set demonstrated substantially better classification performance. Logistic Regression achieved the highest accuracy (0.823 ± 0.077), the highest AUC (0.847 ± 0.099), and the highest MCC (0.663 ± 0.157). SVM-RBF and MLP also showed acceptable performance, with AUC values of 0.737 ± 0.157 and 0.742 ± 0.133, respectively. By comparison, tree-based and ensemble learning methods, including Random Forest, Extra Trees, Gradient Boosting, and LightGBM, performed considerably worse, with AUC values generally ranging from 0.53 to 0.62.

When VEP and MEG features were combined, Logistic Regression remained the best-performing model. It achieved an accuracy of 0.755 ± 0.097, an AUC of 0.819 ± 0.124, and an MCC of 0.532 ± 0.196. Although the combined feature set substantially improved performance compared with the VEP feature set, it did not outperform the MEG feature set alone. No additional performance gain was observed for the remaining machine learning algorithms.

Overall, the MEG feature set achieved the best classification performance for differentiating ON from ION, whereas combining VEP with MEG features did not further improve the results. Logistic Regression was the only algorithm that consistently produced high classification performance (Accuracy = 0.823 ± 0.077, AUC = 0.847 ± 0.099). To statistically compare the discrimination ability of different feature sets, paired bootstrap AUC analysis was additionally performed. The MEG model showed significantly higher AUC than the VEP model (ΔAUC = 0.221, 95% CI: 0.156 to 0.287, *p* < 0.001). Although the VEP–MEG model also achieved significantly higher performance than VEP alone (ΔAUC = 0.179, 95% CI: 0.116 to 0.244, *p* < 0.001), the addition of VEP features did not further improve the MEG-based model (ΔAUC = −0.042, 95% CI: −0.076 to −0.011, *p* = 0.006). The complete statistical comparisons are summarized in Supplementary Table S3.

### 3.4. SHAP Analysis of Feature Importance

To improve the interpretability of the machine learning models, SHAP analysis was performed for the best-performing model of each classification task. Features were ranked according to their mean absolute SHAP values, and the highest-ranked predictors are summarized in Table 4.

**Figure 4:**
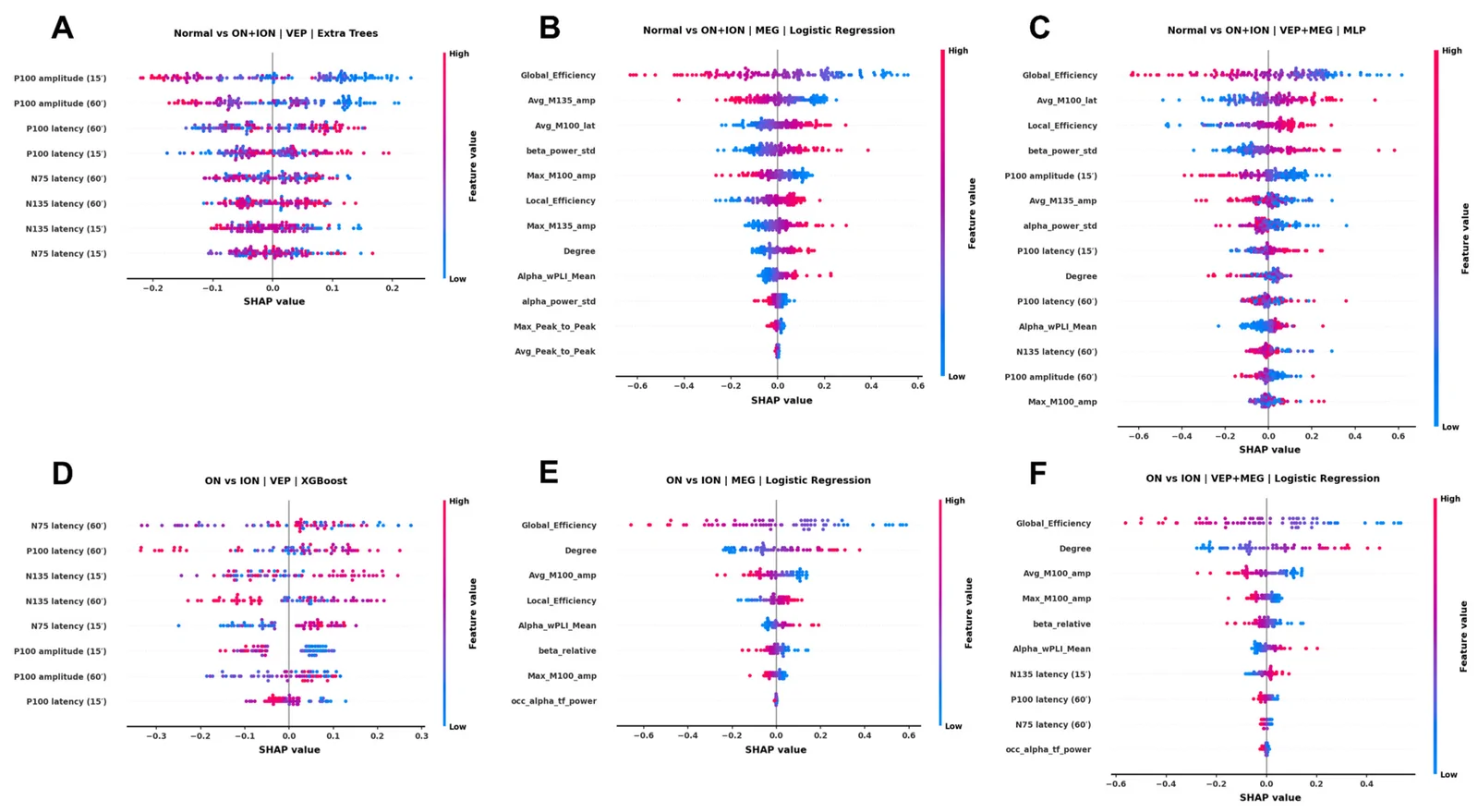
SHAP feature importance analysis of machine learning models. SHAP values were calculated for the best-performing model in each classification task to identify the contribution of individual features to model predictions. Features are ranked according to their mean absolute SHAP values, with higher values indicating greater contribution to classification performance. Panels (**A**–**C**) present SHAP summary plots for the HC vs. ON + ION classification task using the best-performing VEP, MEG, and combined VEP + MEG models, respectively. Panels (**D**–**F**) show the corresponding SHAP analyses for the ON vs. ION classification task. Features are ranked according to their mean absolute SHAP values, with higher-ranked features contributing more strongly to model predictions. Each point represents one sample, and the color indicates the normalized feature value (blue, low; red, high). Positive SHAP values indicate increased contribution toward the positive class, whereas negative SHAP values indicate increased contribution toward the negative class.

For the HC versus ON + ION classification task (Figure 4A–C), VEP feature importance was dominated by the P100 amplitude. The P100 amplitude under the 15′ stimulation condition showed the highest contribution, followed by the P100 amplitude under the 60′ stimulation condition. In comparison, latency-related features contributed less to the model.

The most influential MEG features originated from graph-theoretical metrics, visually evoked responses, and spectral measures. Global Efficiency and Mean Degree ranked highest, followed by maximum M100 amplitude (Max M100 amplitude), average M135 amplitude (Avg M135 amplitude), beta-band power standard deviation, and average M100 latency (Avg M100 latency). Alpha-band functional connectivity (Alpha wPLI) also showed a relatively high contribution. These features consistently ranked among the most important variables in the MEG model.

For the combined feature model, the P100 amplitude remained one of the highest-ranked VEP features. Several MEG features, including Global Efficiency, Mean Degree, Max M100 amplitude, and Alpha wPLI, also appeared among the leading contributors. Together, these results indicate that both VEP and MEG features contributed to model prediction.

For the ON versus ION classification task (Figure 4D–F), the contribution of VEP features was mainly concentrated on the P100 latency, whereas the remaining latency and amplitude features showed relatively limited importance. This ranking was consistent with the relatively modest performance of the VEP-based classification models.

By comparison, the MEG model was primarily driven by graph-theoretical metrics and visually evoked response features. Global Efficiency and Mean Degree consistently ranked among the most important variables. They were followed by Max M100 amplitude, average M100 amplitude (Avg M100 amplitude), occipital alpha-band time–frequency power, Alpha wPLI, and beta-band relative power. These features constituted the major contributors to the MEG classification model.

A similar pattern was observed in the combined feature model. MEG-derived variables remained dominant, with Mean Degree, Global Efficiency, and Max M100 amplitude consistently ranked among the leading features. P100 latency was the highest-ranked VEP feature in the combined model. Overall, graph-theoretical measures and visually evoked MEG responses represented the principal contributors to the discrimination between ON and ION, whereas VEP features provided additional but relatively smaller contributions.

## 4. Discussion

The present study developed machine learning models based on SERF-MEG and VEP to evaluate the diagnostic value of these two neurophysiological techniques for optic neuropathies. Within the framework of this study, three major findings were obtained. First, the combined VEP–MEG feature set achieved the best performance for distinguishing HC from patients with optic neuropathies. Second, MEG features clearly outperformed VEP features in differentiating ON from ION. Third, SHAP analysis showed that the most influential features originated from different dimensions of VEP and MEG across the two classification tasks. These findings indicate that SERF-MEG provides additional cortical neurophysiological information beyond conventional VEP and may serve as a complementary functional assessment tool for the diagnosis and differential diagnosis of optic neuropathies.

For the classification of HC and patients with optic neuropathies, both VEP and MEG achieved satisfactory performance, whereas the combined feature model produced the highest numerical accuracy and AUC. This result suggests that the two techniques capture different aspects of visual system dysfunction and provide complementary information. However, paired bootstrap analysis showed that the improvement in the combined model over the VEP-only model was not statistically significant, suggesting that VEP already contained substantial information for identifying optic neuropathies. Meanwhile, the additional contribution of SERF-MEG may provide complementary cortical functional information beyond conventional VEP measurements. VEP primarily reflects conduction along the visual pathway. By comparison, SERF-MEG measures cortical neuronal activity directly and further characterizes neural oscillations, functional connectivity, and brain network organization. These additional neurophysiological features extend beyond the information provided by conventional VEP. The SHAP analysis supported this interpretation. Both classical VEP parameters and several MEG-derived features ranked highly in the disease classification model, indicating that visual pathway conduction and cortical functional information jointly contributed to model prediction. This finding also explains why the multimodal model outperformed either modality alone.

The most important finding of this study was the superior performance of MEG in differentiating ON from ION. The best MEG model achieved an AUC of 0.847, whereas the best VEP model reached only 0.631. This difference was further supported by paired bootstrap analysis, which demonstrated a significant improvement in MEG-based classification over VEP-based classification (ΔAUC = 0.221, 95% CI: 0.156–0.287, *p* < 0.001). These findings suggest that SERF-MEG captures disease-related cortical functional information that cannot be sufficiently characterized by conventional VEP parameters alone. This difference suggests that conventional VEP parameters alone have limited value for distinguishing these two disorders, while MEG captures additional disease-related neurophysiological information. This difference may be related to the distinct pathological mechanisms underlying ON and ION. Although ON is mainly characterized by inflammatory demyelination and ION by ischemic axonal injury, overlapping axonal degeneration and conduction impairment may occur in both disorders, limiting the diagnostic specificity of conventional VEP measurements.

Recent neuroimaging studies have further shown that optic nerve injury affects not only visual pathway conduction but also cortical function and large-scale visual networks. Altered activation patterns, functional connectivity reorganization, and adaptive cortical plasticity have been reported in patients with ON [32,33]. Different types of optic neuropathies may also produce distinct patterns of cortical functional remodeling. Therefore, although both ON and ION impair visual function, their cortical activity patterns may not be identical. These differences are likely to be reflected by MEG-derived features and may contribute to improved disease classification. The SHAP analysis was consistent with this observation. MEG-derived features contributed more strongly than VEP features in the ON versus ION task, further supporting the additional value of SERF-MEG for disease discrimination. The biological relevance of the SHAP-identified features may be related to the distinct pathophysiological mechanisms underlying ON and ION. Although both diseases cause visual dysfunction, they affect the visual pathway through different primary processes. ON is mainly characterized by inflammatory demyelination, which primarily disrupts signal conduction and may induce adaptive cortical reorganization during disease evolution. In contrast, ION is mainly associated with ischemic injury and axonal loss, which reduces the amount of functional visual input transmitted to the cortex. In this context, M100-related features may reflect differences in cortical responses following visual stimulation. Alterations in M100 amplitude may represent variations in preserved afferent input and cortical responsiveness caused by different patterns of optic nerve damage. Meanwhile, graph-theoretical measures, including Global Efficiency and Mean Degree, may capture differences in large-scale visual network organization after injury. The distinct balance between functional preservation and cortical adaptation in ON and ION may result in different patterns of network integration, which could be detected by these measures. Alpha wPLI provides additional information regarding inter-regional synchronization. Changes in alpha-band connectivity may reflect differences in cortical communication and compensatory network remodeling between inflammatory and ischemic optic nerve injuries. Therefore, the superior contribution of MEG-derived features in ON versus ION classification may result from its ability to capture not only the final output of visual pathway dysfunction but also the complex cortical functional changes associated with different disease mechanisms.

An interesting finding was that combining VEP with MEG features did not further improve classification performance over the MEG model alone. One possible explanation is that the additional information provided by VEP was limited after the MEG characteristics had already been integrated. Another hypothesis is that the very small sample size diminished the advantage of adding new characteristics and hindered model generalizability. Similar findings have been reported in other medical machine learning studies, where incorporating additional features does not necessarily increase classification accuracy when the new information is redundant or just weakly useful.

Another observation deserves attention. Logistic Regression consistently achieved the best performance for differentiating ON from ION, whereas most nonlinear and ensemble learning models performed considerably worse. This result may indicate that the discriminative information between the two diseases can be captured using a relatively simple decision boundary. Alternatively, the poorer performance of more complex models may reflect the limited sample size, which increases model variance and reduces generalizability. At present, these findings should be interpreted with caution. Larger multicenter datasets will be required to determine whether this pattern remains stable across independent cohorts.

Compared with previous studies that mainly focused on time-domain MEG evoked responses, this study integrated frequency-domain, functional connectivity, and graph-theoretical features into the machine learning framework. This strategy provided a more comprehensive description of visual system function. Within the framework of this study, these features were not intended to replace conventional time-domain measures such as the M100 and M135 responses. Instead, they served as complementary biomarkers and supplied additional discriminative information for model prediction. Because the sample size was limited, these findings should still be regarded as exploratory. Their clinical value needs to be confirmed in larger independent cohorts.

The overall findings of this study suggest that SERF-MEG has the potential to complement conventional VEP in the evaluation of optic neuropathies. Additional functional information may be provided for both disease detection and the differential diagnosis of optic neuropathies with different etiologies. Recent advances in SERF atomic magnetometer technology have improved the feasibility of clinical MEG applications. Unlike conventional superconducting MEG systems, SERF-MEG does not require liquid helium cooling and provides a more scalable platform for future clinical translation. Previous OPM-MEG and SERF-MEG studies have mainly focused on demonstrating non-cryogenic magnetic recording and characterizing sensory-evoked cortical responses. The present study extends these efforts by applying SERF-MEG to optic neuropathies and integrating multidimensional MEG features with machine learning for diagnosis and differential diagnosis.

Beyond diagnostic performance, several practical considerations should be addressed for future clinical translation. The current SERF-MEG examination can be completed within approximately 10 min, and unlike VEP, it does not require electrode placement or impedance checking, which may simplify patient preparation. In addition, SERF-MEG provides flexible paradigms beyond conventional visual evoked responses, allowing for the future investigation of broader cortical visual functions. Although model development involves computationally intensive procedures, prediction after model establishment is relatively lightweight and may support clinical implementation. However, SERF-MEG remains an emerging technology with limited clinical availability, and further multicenter validation and workflow optimization are required. In clinical practice, SERF-MEG is not intended to replace VEP but may provide complementary functional information with machine learning-assisted interpretation.

Several limitations should also be acknowledged. First, the sample size was relatively small, particularly in the subgroup analysis of ON and ION. This limitation may have affected model stability and generalizability, especially for disease subtype classification with relatively subtle biological differences. Although nested cross-validation, bootstrap confidence interval estimation, and feature selection robustness analysis were implemented to reduce overfitting and evaluate model stability, these approaches cannot completely eliminate the uncertainty associated with a limited sample size. Therefore, the current findings, particularly those related to ON versus ION classification, should be considered exploratory and require further validation in larger, multicenter cohorts. Second, this was a single-center study based on a single SERF-MEG system, and no independent external validation cohort was available. Because SERF-MEG is an emerging technology with limited availability across clinical centers, external validation in larger multicenter cohorts remains necessary to confirm the generalizability and clinical applicability of the proposed models. In addition, differences in MEG systems, acquisition protocols, and preprocessing procedures may affect model performance. The applicability of this framework to other MEG platforms, including conventional superconducting MEG systems, requires further investigation. Third, only conventional supervised machine learning algorithms were evaluated in this study. The analysis was mainly based on SERF-MEG and VEP electrophysiological features, while other clinically relevant information, including OCT measurements, visual field data, and routine clinical variables, was not incorporated. Future research should integrate structural imaging, electrophysiological measures, and clinical factors into multimodal diagnostic models to further evaluate the clinical value of SERF-MEG.

## 5. Conclusions

Overall, this study illustrates the viability of integrating SERF-MEG into machine learning models for the diagnosis of optic neuropathies. SERF-MEG provides complementary neurophysiological information beyond that received from standard VEP. It also enhances the differentiation of optic neuropathies with diverse etiologies. Although these findings require validation in larger multicenter cohorts, this work provides preliminary support for the future clinical translation of SERF-MEG as a supplemental neurophysiological tool for the diagnosis and differential diagnosis of neuro-ophthalmic disorders.

## Figures and Tables

**Table 1:** Clinical characteristics of study participants.

Characteristic	HC	ON	ION
Participants	71	34	37
Age	46.3 ± 11.3	45.8 ± 11.5	53.1 ± 12.5
Male/Female	29/42	13/21	22/15
Best-corrected visual acuity (logMAR)	0.00 ± 0.05	0.35 ± 0.45	0.55 ± 0.52
Previous treatment	NA	28 (82.4%)	25 (67.6%)

Values are presented as mean ± standard deviation or number (%). NA, not applicable.

**Table 2:** Classification performance for normal controls versus optic neuropathy.

Feature Set	Model	Accuracy	AUC	F1	Sensitivity	Specificity	MCC
VEP	Logistic Regression	0.673 ± 0.075	0.769 ± 0.086	0.688 ± 0.080	0.732 ± 0.132	0.614 ± 0.125	0.358 ± 0.155
	SVM (RBF)	0.648 ± 0.064	0.665 ± 0.208	0.674 ± 0.077	0.749 ± 0.157	0.545 ± 0.171	0.317 ± 0.121
	Random Forest	0.708 ± 0.083	0.769 ± 0.066	0.704 ± 0.096	0.714 ± 0.153	0.701 ± 0.115	0.427 ± 0.172
	Gradient Boosting	0.670 ± 0.088	0.737 ± 0.091	0.649 ± 0.117	0.632 ± 0.155	0.707 ± 0.121	0.346 ± 0.177
	**Extra Trees**	**0.692 ± 0.078**	**0.779 ± 0.070**	**0.689 ± 0.095**	**0.700 ± 0.140**	**0.684 ± 0.112**	**0.392 ± 0.156**
	AdaBoost	0.661 ± 0.097	0.724 ± 0.089	0.661 ± 0.103	0.669 ± 0.136	0.653 ± 0.129	0.328 ± 0.197
	MLP	0.687 ± 0.082	0.741 ± 0.074	0.673 ± 0.105	0.664 ± 0.154	0.711 ± 0.106	0.383 ± 0.166
	XGBoost	0.687 ± 0.080	0.743 ± 0.078	0.669 ± 0.095	0.646 ± 0.137	0.727 ± 0.144	0.386 ± 0.169
	LightGBM	0.707 ± 0.069	0.745 ± 0.080	0.702 ± 0.077	0.706 ± 0.135	0.707 ± 0.126	0.426 ± 0.143
MEG	Logistic Regression	0.665 ± 0.084	0.733 ± 0.093	0.664 ± 0.090	0.671 ± 0.133	0.660 ± 0.146	0.342 ± 0.177
	SVM (RBF)	0.643 ± 0.071	0.674 ± 0.148	0.647 ± 0.073	0.661 ± 0.112	0.625 ± 0.132	0.293 ± 0.145
	Random Forest	0.648 ± 0.103	0.720 ± 0.101	0.639 ± 0.120	0.643 ± 0.168	0.654 ± 0.132	0.306 ± 0.213
	Gradient Boosting	0.647 ± 0.092	0.705 ± 0.104	0.641 ± 0.105	0.643 ± 0.140	0.651 ± 0.125	0.299 ± 0.187
	Extra Trees	0.641 ± 0.089	0.711 ± 0.096	0.645 ± 0.098	0.667 ± 0.140	0.615 ± 0.115	0.289 ± 0.182
	AdaBoost	0.624 ± 0.079	0.685 ± 0.075	0.621 ± 0.096	0.629 ± 0.134	0.620 ± 0.100	0.252 ± 0.159
	**MLP**	**0.675 ± 0.090**	**0.714 ± 0.105**	**0.670 ± 0.098**	**0.673 ± 0.138**	**0.678 ± 0.130**	**0.359 ± 0.186**
	XGBoost	0.634 ± 0.087	0.703 ± 0.092	0.623 ± 0.109	0.624 ± 0.154	0.645 ± 0.101	0.273 ± 0.175
	LightGBM	0.625 ± 0.089	0.687 ± 0.102	0.618 ± 0.107	0.623 ± 0.152	0.629 ± 0.133	0.261 ± 0.185
VEP + MEG	Logistic Regression	0.679 ± 0.073	0.777 ± 0.073	0.682 ± 0.081	0.698 ± 0.117	0.658 ± 0.133	0.363 ± 0.147
	SVM (RBF)	0.693 ± 0.079	0.763 ± 0.079	0.694 ± 0.086	0.706 ± 0.122	0.679 ± 0.110	0.391 ± 0.159
	Random Forest	0.717 ± 0.066	0.781 ± 0.070	0.711 ± 0.070	0.704 ± 0.112	0.729 ± 0.128	0.443 ± 0.133
	Gradient Boosting	0.673 ± 0.076	0.746 ± 0.077	0.662 ± 0.082	0.650 ± 0.122	0.695 ± 0.140	0.354 ± 0.152
	Extra Trees	0.705 ± 0.090	0.776 ± 0.080	0.701 ± 0.094	0.695 ± 0.120	0.715 ± 0.135	0.418 ± 0.182
	AdaBoost	0.666 ± 0.065	0.731 ± 0.081	0.653 ± 0.079	0.639 ± 0.121	0.693 ± 0.114	0.339 ± 0.132
	**MLP**	**0.732 ± 0.066**	**0.792 ± 0.069**	**0.725 ± 0.074**	**0.713 ± 0.097**	**0.752 ± 0.096**	**0.470 ± 0.133**
	XGBoost	0.689 ± 0.066	0.757 ± 0.082	0.666 ± 0.078	0.631 ± 0.118	0.746 ± 0.126	0.388 ± 0.133
	LightGBM	0.675 ± 0.107	0.747 ± 0.099	0.662 ± 0.120	0.650 ± 0.151	0.701 ± 0.152	0.358 ± 0.217

**Table 3:** Classification performance for the differential diagnosis of ON and ION.

Feature Set	Model	Accuracy	AUC	F1	Sensitivity	Specificity	MCC
VEP	Logistic Regression	0.475 ± 0.116	0.488 ± 0.142	0.472 ± 0.161	0.484 ± 0.210	0.464 ± 0.196	−0.060 ± 0.246
	SVM (RBF)	0.555 ± 0.107	0.560 ± 0.138	0.565 ± 0.120	0.576 ± 0.180	0.530 ± 0.202	0.115 ± 0.223
	Random Forest	0.597 ± 0.107	0.616 ± 0.143	0.624 ± 0.120	0.667 ± 0.189	0.515 ± 0.220	0.194 ± 0.243
	Gradient Boosting	0.561 ± 0.092	0.572 ± 0.126	0.595 ± 0.102	0.638 ± 0.165	0.478 ± 0.174	0.125 ± 0.189
	Extra Trees	0.580 ± 0.106	0.601 ± 0.134	0.609 ± 0.113	0.652 ± 0.189	0.498 ± 0.234	0.167 ± 0.241
	AdaBoost	0.522 ± 0.126	0.555 ± 0.143	0.531 ± 0.139	0.541 ± 0.189	0.501 ± 0.188	0.050 ± 0.259
	MLP	0.552 ± 0.142	0.558 ± 0.132	0.554 ± 0.165	0.572 ± 0.229	0.530 ± 0.205	0.108 ± 0.300
	**XGBoost**	**0.614 ± 0.082**	**0.631 ± 0.134**	**0.630 ± 0.090**	**0.648 ± 0.151**	**0.576 ± 0.160**	**0.236 ± 0.174**
	LightGBM	0.566 ± 0.121	0.552 ± 0.145	0.581 ± 0.114	0.583 ± 0.140	0.546 ± 0.197	0.133 ± 0.255
MEG	**Logistic Regression**	**0.823 ± 0.077**	**0.847 ± 0.099**	**0.833 ± 0.070**	**0.845 ± 0.115**	**0.802 ± 0.151**	**0.663 ± 0.157**
	SVM (RBF)	0.705 ± 0.116	0.737 ± 0.157	0.719 ± 0.113	0.729 ± 0.143	0.685 ± 0.187	0.424 ± 0.235
	Random Forest	0.569 ± 0.120	0.563 ± 0.165	0.607 ± 0.116	0.651 ± 0.164	0.482 ± 0.184	0.139 ± 0.253
	Gradient Boosting	0.564 ± 0.159	0.594 ± 0.206	0.584 ± 0.160	0.604 ± 0.202	0.526 ± 0.199	0.136 ± 0.327
	Extra Trees	0.555 ± 0.134	0.567 ± 0.165	0.580 ± 0.139	0.611 ± 0.188	0.494 ± 0.212	0.109 ± 0.280
	AdaBoost	0.609 ± 0.133	0.624 ± 0.150	0.630 ± 0.130	0.651 ± 0.178	0.569 ± 0.230	0.226 ± 0.279
	MLP	0.716 ± 0.116	0.742 ± 0.133	0.738 ± 0.108	0.770 ± 0.140	0.658 ± 0.193	0.441 ± 0.237
	XGBoost	0.584 ± 0.127	0.617 ± 0.151	0.603 ± 0.128	0.624 ± 0.177	0.543 ± 0.200	0.170 ± 0.260
	LightGBM	0.515 ± 0.108	0.529 ± 0.169	0.515 ± 0.148	0.531 ± 0.209	0.500 ± 0.200	0.035 ± 0.240
VEP + MEG	**Logistic Regression**	**0.755 ± 0.097**	**0.819 ± 0.124**	**0.760 ± 0.097**	**0.754 ± 0.147**	**0.761 ± 0.177**	**0.532 ± 0.196**
	SVM (RBF)	0.606 ± 0.084	0.660 ± 0.127	0.620 ± 0.098	0.641 ± 0.173	0.572 ± 0.211	0.225 ± 0.177
	Random Forest	0.535 ± 0.136	0.577 ± 0.145	0.556 ± 0.172	0.600 ± 0.226	0.465 ± 0.192	0.062 ± 0.283
	Gradient Boosting	0.541 ± 0.118	0.594 ± 0.149	0.561 ± 0.130	0.581 ± 0.177	0.494 ± 0.215	0.073 ± 0.254
	Extra Trees	0.554 ± 0.118	0.556 ± 0.143	0.592 ± 0.115	0.634 ± 0.171	0.470 ± 0.224	0.102 ± 0.254
	AdaBoost	0.549 ± 0.111	0.582 ± 0.096	0.556 ± 0.135	0.569 ± 0.189	0.530 ± 0.203	0.097 ± 0.243
	MLP	0.608 ± 0.092	0.649 ± 0.131	0.613 ± 0.114	0.626 ± 0.182	0.596 ± 0.184	0.234 ± 0.184
	XGBoost	0.580 ± 0.096	0.615 ± 0.100	0.594 ± 0.113	0.616 ± 0.185	0.544 ± 0.181	0.171 ± 0.206
	LightGBM	0.520 ± 0.090	0.504 ± 0.136	0.547 ± 0.084	0.566 ± 0.126	0.470 ± 0.208	0.032 ± 0.201

**Table 4:** Top-ranked features identified by SHAP analysis.

Task	Rank	Feature	Category	Selection Frequency (%)	Importance Score
Normal vs. ON + ION	1	P100 amplitude (15′)	VEP	100	2.3056
	2	Global efficiency	Graph	100	1.8659
	3	Degree	Graph	76	0.9663
	4	Max M100 amplitude	Temporal	100	0.4857
	5	Alpha wPLI mean	Connectivity	76	−0.4554
	6	Average M135 amplitude	Temporal	96	0.4311
	7	Alpha power SD	Spectral	72	0.4049
	8	P100 amplitude (60′)	VEP	100	0.3962
	9	Local efficiency	Graph	84	0.2905
	10	Average M100 latency	Temporal	100	0.1943
ON vs. ION	1	Global efficiency	Graph	100	1.6318
	2	Degree	Graph	100	1.5961
	3	Max M100 amplitude	Temporal	100	1.0123
	4	P100 latency (60′)	VEP	100	0.8759
	5	Average M100 amplitude	Temporal	64	0.5995
	6	Occipital alpha TF power	Spectral	36	0.5510
	7	N135 latency (15′)	VEP	100	0.4120
	8	Beta relative power	Spectral	48	0.3081
	9	Local efficiency	Graph	44	0.3068
	10	Alpha wPLI mean	Connectivity	56	0.2140

## Data Availability

The data presented in this study are available on request from the corresponding author. The data are not publicly available due to patient privacy and ethical restrictions.

## Supplementary Materials

The following supporting information can be downloaded at: https://www.mdpi.com/article/10.3390/bioengineering13080885/s1, Table S1: Comparison of feature selection strategies; Table S2A: Hyperparameter search space and number of evaluated combinations for machine learning classifiers; Table S2B: Most frequently selected hyperparameter combinations during nested cross-validation; Table S3: Paired bootstrap comparison of AUC differences between machine learning models.

## Abbreviations

The following abbreviations are used in this manuscript:

AUC	Area Under the Receiver Operating Characteristic Curve
Avg	Average method
HC	Healthy Controls
ICA	Independent component analysis
ION	Ischemic optic neuropathy
ISCEV	International Society for Clinical Electrophysiology of Vision
LightGBM	Light Gradient Boosting Machine
MCC	Matthews Correlation Coefficient
MEG	Magnetoencephalography
MLP	Multi-layer Perceptron
ON	Optic neuritis
PSD	Power Spectral Density
ROI	Region of Interest
SERF	Spin-exchange relaxation-free
SERF-MEG	Spin-exchange relaxation-free magnetoencephalography
SHAP	Shapley Additive Explanations
SMOTE	Synthetic Minority Over-sampling Technique
VEF	Visual evoked fields
VEP	Visual evoked potentials
wPLI	Weighted Phase Lag Index
XGBoost	Extreme Gradient Boosting
